# Physiological Toxicity and Antioxidant Mechanism of Photoaging Microplastics on *Pisum sativum* L. Seedlings

**DOI:** 10.3390/toxics11030242

**Published:** 2023-03-04

**Authors:** Mengen Kang, Yi Liu, Haoke Wang, Yuzhu Weng, Dongqing Gong, Xue Bai

**Affiliations:** 1 Key Laboratory of Integrated Regulation and Resource Development on Shallow Lake of Ministry of Education, College of Environment, Hohai University, Nanjing 210098, China; 2 Yangtze Institute for Conservation and Development, Hohai University, Nanjing 210098, China

**Keywords:** microplastics, photoaging, phytotoxicity, reactive oxygen species, antioxidant system

## Abstract

Recent studies have confirmed that changes in the physical properties of microplastics (MPs) trigger toxicological effects and ecological risks. To explore the toxicity of different types of MPs on plants, and the influence of MP photoaging, this study investigated the toxicity mechanisms of pristine, 7 and 14 d photoaged polystyrene (PS), polyamide (PA), polyethylene (PE), and polyethylene terephthalate (PET) MPs on seed germination, root growth, nutrient fraction, oxidative stress, and antioxidant systems of *Pisum sativum* L. (pea) seedlings. The results showed that pristine PS and 14 d photoaged PET inhibited seed germination. Compared to the pristine MPs, photoaged MPs had negative effects on root elongation. Moreover, photoaged PA and PE impeded the nutrient transport of soluble sugars from roots to stems. Notably, the production of superoxide anion radicals (•O_2_^−^) and hydroxyl radicals (•OH) through the photoaging of MPs exacerbated oxidative stress and reactive oxygen species formation in roots. Antioxidant enzyme data revealed that the activities of superoxide dismutase and catalase were significantly activated in photoaged PS and PE, respectively, in order to scavenge •O_2_^−^ and hydrogen peroxide (H_2_O_2_) accumulation and alleviate lipid peroxidation levels in cells. These findings provide a new research perspective on the phytotoxicity and ecological risk of photoaged MPs.

## 1. Introduction

With the massive production and consumption of plastic, these products are becoming a significant pollutant in the environment. Bulk plastic can be degraded into microplastics (MPs) smaller than 5 mm in size under physical, chemical, and biological forces [1]. MPs have become an essential object of research concern in recent years, and this emerging contaminant has been detected in freshwater [2], marine systems [3], air [4], and soil [5]. There is a wide variety in the types of MPs detected in soil and water in China and abroad [6]; the MPs were detected in rivers, lakes, and estuaries in Asia, Australia, and North America at concentrations ranging from 1 × 10^−2^ to 10^8^ particles/m^3^ [7], with polypropylene (PP) and polyethylene (PE) being the most abundant in freshwater [8], while three types of MPs found at high levels in agricultural fields in southeastern Germany were PE, polystyrene (PS) and PP [6]. Meanwhile, the primary components of MPs in agricultural soils in Hangzhou Bay, Zhejiang Province, were PE, PP, polyethylene terephthalate (PET), acrylic acid, and polyamide (PA) [9], while large amounts of PP, PS, and PE were identified in the Danjiangkou Reservoir [10]. Various types of MPs may lead to differences in shape, size, flexibility, and chemical composition, resulting in different ecotoxicity effects [11]. Studies have indicated that polyvinyl chloride (PVC) treatment is able to significantly raise aminolevulinic acid and proline levels in *Lepidium sativum* (garden cress) compared to PP and PE [12], causing powerful toxicological effects. It has also been confirmed [13] that both low-density polyethylene (LDPE) and biodegradable plastics could affect *Triticum aestivum* L. (common wheat) growth and that the negative impact of biodegradable plastics was stronger than that of LDPE. Similarly, different concentrations of PS, PE, and PP MPs all produced phytotoxicity resulting in growth inhibition and oxidative stress in *Lycopersicon esculentum* L. (tomato), and PE exhibited greater physiological toxicity on seedlings than PS and PP [14]. Therefore, it is essential to clarify the biological toxicity differences and modulation mechanisms of different types of MPs for a comprehensive understanding of the ecological risks and toxicity effects of particles.

Photoaging is a quite common and typical behavior of MPs in the environment, which may lead to significant changes in their particle properties and structures [15]. The surface of MPs under photoirradiation often becomes rough, cracked, embrittled, flaky, and disintegrated [16] and even leads to changes in a specific surface area to some extent. In addition, it has been suggested [17] that photoaging can induce the production of environmentally persistent free radicals and reactive oxygen species (ROS) in MPs. These highly reactive species have a strong oxidative capacity and may be able to influence associated biotoxicity. Some studies have confirmed the ability of MPs to induce oxidative stress in plants [18,19]. For instance, PS MPs were shown to be able to induce chromosomal abnormalities and genotoxicity in *Allium cepa* L. (onion) cells by increasing the generation of ROS [20]. Additionally, the oxidative stress response of organisms is usually due to the imbalance of ROS levels in the body. Notably, it was hypothesized that there may be a correlation between the activated species formed by the photoaging of MPs and their biological effects. Previous studies have also proposed [15,21] that the changes caused by the aging of MPs in the environment may be related to the type and shape of the plastic and the additives used in the processing, indicating that the photoaging process of MPs may be influenced by physicochemical properties. Therefore, it is more critical and appropriate to focus on the effects of MP types in conjunction with their possible photoaging behaviors in the environment and to further model the biological toxicity of particles in the natural environment.

In this study, in order to investigate the physiological toxicity and modulation mechanisms of photoaging of different types of MPs on *Pisum sativum* L. (pea) seedlings, four kinds of common MPs, namely PS, PA, PE, and PET MPs, were selected as experimental materials. Specifically, this study (1) assessed the levels of ROS generated by the photoaging of different types of MPs under simulated sunlight irradiation; (2) detected the effects of MPs on seed germination, root elongation, stem length, lateral root number, and soluble sugar content in seedlings; and (3) investigated the oxidative stress and antioxidant regulation mechanisms induced by treatments of MP exposure in roots. This study will contribute to a better insight into the potential mechanisms of photoaging MP-induced phytotoxicity.

## 2. Materials and Methods

### 2.1. Experimental Materials

The MPs used were purchased from Zhonglian Plastic Chemical Company Limited (Guangdong, China). Particles sized 100 μm were chosen, and PS, PA, PE, and PET MPs were selected as the experimental materials.

### 2.2. Light Aging Irradiation Test

The MP powders were artificially aged in a light simulation chamber (Q-SUN XE-1, Q-LAB, Llantrisant, UK). Samples of 25 g of PS, PA, PE, and PET MPs were placed uniformly in Petri dishes in a light simulation chamber with a UV wavelength of 340 nm, room temperature of 35 °C, and irradiance of 0.51 W/m^2^. The four types of MPs were sampled separately at the indicated periods (7 and 14 d).

### 2.3. Superoxide Anions and Hydroxyl Radicals in MP Suspensions

The concentrations of superoxide anion radicals (•O_2_^−^) and hydroxyl radicals (•OH) were determined by dispersing 100 mg of pristine, 7 and 14 d photoaged samples of PS, PA, PE, and PET MPs in 1 L of distilled water. The mixture was well shaken at 70 rpm at 37 °C and then filtered through an aqueous 0.45 μm microporous filter membrane. The •O_2_^−^ is able to reduce nitro blue tetrazolium (NBT) to blue methylhydrazone, which has a maximum light absorption at 560 nm, thus indirectly measuring the superoxide anion radical content. For each group of filtrate with 0.1 mM of NBT, the absorbance values were obtained at 560 nm after incubation in the dark for 30 min with ultrapure water as the control group.

The •OH are strong oxidants that can react with coumarin to form the strongly fluorescent substance 7-hydroxycoumarin. The fluorescence intensity of 7-hydroxycoumarin was directly correlated with the concentration of •OH. A standard curve was created with the standard solution of 7-hydroxycoumarin in the dose range of 0–10 μM. Subsequently, the 0.1 mM coumarin was added to each group of filtrate separately, and the fluorescence intensity was measured at the excitation wavelength of 332 nm and emission wavelength of 455 nm to determine the concentration of •OH.

### 2.4. Identification of MPs by Fourier Transform Infrared Microscopy

To evaluate the possible surface changes of MPs after photoaging, four types of MPs were measured in the wavelength range 4000–650 cm^−1^ using Fourier transform infrared microscopy spectroscopy (FTIR, Spotlight 200i, PerkinElmer, OH, USA) for both pristine MPs and differently photoaged MPs.

### 2.5. Seedling Culture and Treatments

*Pisum sativum* L. seeds were first disinfected with 0.02% NaClO solution for 20 min, rinsed three times with ultrapure water, soaked in warm water at 55 °C for 10 min, and finally immersed in cold water for 12 h. After sufficient soaking, full and evenly sized seeds were selected for cultivation in Petri dishes.

Seeds from each group of 50 were incubated in 100 mg L^−1^ of pristine, 7 and 14 d photoaged suspensions of PS, PA, PE, and PET MPs for three replicates and germinated at 20–25 °C. Then the germination rate of seedlings was recorded after 48 h.

For the formal exposure test, full and uniformly sized seeds were placed in ultrapure water and germinated at 20–25 °C. The seedlings with similar germination status were selected for hydroponics in 100 mg L^−1^ pristine and photoaging suspensions with three replicates of 30 plants each, for a total of 90 plants. The artificial climate chamber was set at 22 and 18 °C d/night for 16 and 8 h d/night durations, respectively. Toxicity endpoints were measured after 7 d of growth, including root elongation, stem length, and the number of lateral roots for each group of seedlings.

### 2.6. Physiological and Biochemical Analysis of Seedlings

#### 2.6.1. Soluble Sugar Content Detection

The soluble sugar content in the root and stem tissues was determined using the phenol method. From the roots and above-ground parts from each group after 7 d of MP exposure treatment, 0.3 g were taken, put into graduated test tubes, mixed with 5–10 mL of ultrapure water, and sealed with plastic film. Subsequently, the mixture was soaked in boiling water for 30 min and extracted twice, and the extraction solution was filtered into 25 mL volumetric flasks. The tubes and residues were rinsed repeatedly, and the volume was fixed with ultrapure water. Immediately afterward, 0.5 mL of the sample solution was pipetted into the tube along with 1.5 mL of ultrapure water, and 1 mL of 9 g mL^−1^ phenol solution was added sequentially to the tube and shaken well. Finally, the absorbance was measured at 485 nm by adding 3 mL of concentrated sulfuric acid and reacting fully at room temperature for 30 min.

#### 2.6.2. Lipid Peroxidation Testing

To verify the cell membrane integrity of root tissues, the roots of each group treated with MP exposure for 7 d were rinsed repeatedly in ultrapure water and dried, then immersed in 0.25% Evans blue staining solution. Followed immediately by shaking in a 100 rpm shaker for 30 min, the staining was washed with ultrapure water for 5 min, which was repeated 3 times, and the excess stain was removed. The samples were dried and photographed to record the staining of roots.

The malondialdehyde (MDA) content was measured [22] to assess the lipid peroxidation level in plant cells. Briefly, the 0.3 g of fresh root tissues were ground in an ice bath and centrifuged for 15 min at 12,000 rpm after making a homogenate in 6 mL of phosphate-buffered solution (PBS 50 mM, pH 7.8). Then, 5% trichloroacetic acid (TCA) and 5% thiobarbituric acid (TBA) were mixed with the enzyme extract and heated in a water bath for 20 min. The absorbance of the supernatants were measured at 532 and 600 nm after centrifugation at 4800 rpm for 10 min. The electrolyte leakage rate (ELR) was determined using the conductivity method and expressed as relative conductivity.

#### 2.6.3. Detection of Reactive Oxygen Content

The •O_2_^−^ and hydrogen peroxide (H_2_O_2_) are essential reactive oxygen substances in the course of biological physiological activities and are used as important indicators of reactive oxygen content in roots [23]. The 0.5 g of each group of root samples was weighed in 0.05 M PBS (pH 7.8) and homogenized. Then, the homogenate was centrifuged at 3000 rpm for 10 min and 2 mL of supernatant was mixed with 1.5 mL of PBS and 0.5 mL of 0.01 M hydrogenamine hydrochloride followed by incubation in water at 25 °C for 10 min. Subsequently, the mixture was added with 2 mL of 17 mM sulfonamide and 7 mM α-naphthylamine and incubated at 30 °C for 20 min. The absorbance was recorded at 530 nm to calculate the concentration of •O_2_^−^.

Root tissues treated with MP exposure were weighed 0.1 g and homogenized in 0.1 M PBS (pH 7.0). The mixture was transferred to 1.5 mL of 0.1% TCA and centrifuged at 4000 rpm for 15 min. Next, 1.5 mL of supernatant was added to 0.5 mL of 0.1 M PBS and 1 mL of 1 M potassium iodide. Lastly, the concentration of H_2_O_2_ was determined by sufficient shaking for 1 h at 28 °C and reading the absorbance at 390 nm.

#### 2.6.4. Antioxidant Enzyme Activity Assay

Seedling roots were extracted for the analysis of antioxidant enzyme activity. The frozen 0.3 g of tissue was ground using a pre-chilled mortar and homogenized with 6 mL of 65 mM PBS (pH 7.0). The tissues were then centrifuged at 12,000 rpm for 15 min. As described in a previous study, the supernatant was used to determine superoxide dismutase (SOD) and catalase (CAT) [23].

### 2.7. Data Analysis

A one-way analysis of variance (ANOVA) was performed on the data using SPSS software. The results of three replicates were expressed as the mean ± standard deviation (SD). Data were considered statistically significant only when *p* < 0.05.

## 3. Results and Discussion

### 3.1. Free Radical Generation in Photoaging MP Suspensions

**Figure 1:**
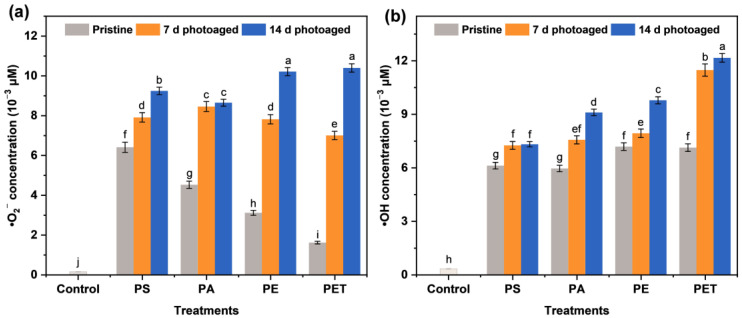
The production of (**a**) superoxide anion radicals (•O_2_^−^) and (**b**) hydroxyl radicals (•OH) in suspensions of different microplastics (MPs) of polystyrene (PS), polyamide (PA), polyethylene (PE), and polyethylene terephthalate (PET) after 0, 7, and 14 d of photoaging. The number of samples for the repeated experiments is *n* = 3. Data are expressed as the mean ± standard deviation (SD). Different lowercase letters indicate significant differences between experimental groups (*p* < 0.05).

According to Figure 1, it can be seen that the amount of ROS generation in MP suspensions is highly associated with the type of particles. Different types of MPs exhibited a significant difference in their ability to generate •O_2_^−^ in a pristine state, while their capacity to produce •OH showed no significant variation. The initial PS MPs were observed to create high levels of •O_2_^−^ and •OH in suspension, reaching 6.43 and 6.12 μM L^−1^, respectively. With the photoaging treatment of the MPs, the production of both •O_2_^−^ and •OH was obviously enhanced. The previous study [24] also confirmed that photoaging was able to increase the adsorption behavior and ROS generation of MPs. In particular, the enhancement of ROS production varied with photoaging time in all four types of MP suspensions. The PET MPs photoaged for 14 d were able to trigger the generation of 10.41 and 12.17 μM L^−1^ •O_2_^−^ and •OH, respectively. These results indicate that photo-irradiation can induce ROS generation in MP suspensions, and this effect varies for different types of MPs, probably due to the physicochemical properties of the particles.

### 3.2. The FTIR Analysis of Photoaging MPs

**Figure 2:**
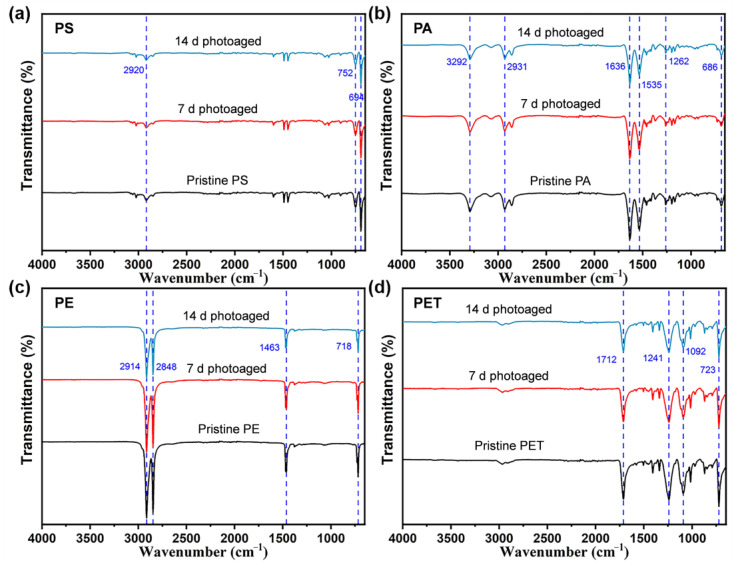
Fourier transform infrared spectra (4000–650 cm^−1^) of (**a**) PS, (**b**) PA, (**c**) PE, and (**d**) PET MPs after photo-irradiation for 0, 7, and 14 d. The number of samples for the repeated experiments is *n* = 3.

To further understand the effect of photoaging of MPs, a FTIR analysis of four types of MPs was performed on four types of MPs at different photoaging times in the experiment (Figure 2). The FTIR spectra showed that photoaging treatment did not significantly influence the surface chemistry of MPs and no new substances were generated during the process. This is different from the results of some academics [25,26], who found that photo-irradiation was able to induce the formation of oxygen-containing functional groups on the surface of PS MPs, which may be due to the relatively short time of photoaging of MPs or the low radiation intensity in this study. However, it is consistent that four types of MPs showed a significant decrease in the intensity of some characteristic peaks after photo-irradiation exposure. More specifically, the absorption peaks at 2920, 752, and 694 cm^−1^ in the FTIR spectra of PS MPs are the C–H and C=C stretching vibration peaks of hydrocarbons, respectively, and the intensity of these absorption peaks decrease with increasing photoaging time. Similarly, the PE MPs showed a weakening of the peak intensities of C–H (2914, 2848, and 1463 cm^−1^) and C=C (718 cm^−1^). The PA MPs also exhibited a decrease in the peak intensities of C–H (3292 and 2931 cm^−1^), N–H (1636 cm^−1^), N–O (1535 cm^−1^), C–O (1262 cm^−1^), and C=C (686 cm^−1^) with the duration of light intensity. The FTIR spectra of PET MPs were attenuated at 1712 cm^−1^ (C=O), 1241 and 1092 cm^−1^ (C–O), and 723 cm^−1^ (C=C) peaks. Collectively, this suggests that the weak binding bonds of the MPs may break under simulated natural light irradiation with photoaging induction, which may lead to the oxidation of the MP surface and thus the ROS formation in the suspension.

### 3.3. Seedling Germination and Physiological Status

**Figure 3:**
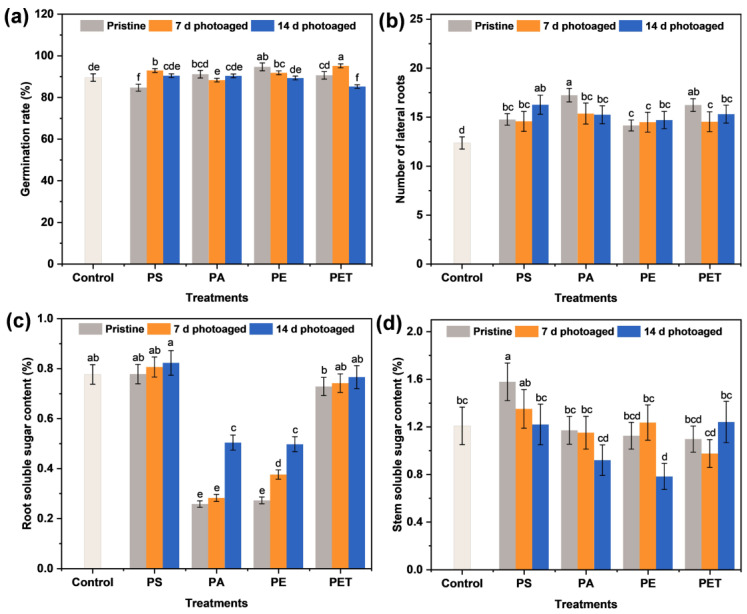
The (**a**) germination rate of sprouts, (**b**) the number of lateral roots, and the soluble sugar content in (**c**) roots and (**d**) stems after 7 d of exposure to 100 mg L^−1^ different MP suspensions. The number of samples for the repeated experiments is *n* = 3. Data are expressed as the mean ± SD. Different lowercase letters indicate significant differences between experimental groups (*p* < 0.05).

To evaluate the effects of treatments of the four types of photoaged MPs on the sprout growth, the physiological morphology, germination rate, root elongation, stem length, and the number of lateral roots were observed and recorded after each group of particle exposure. It can be seen that there was no significant impact of each group of MPs on the germination of seeds (Figure 3a). In fact, after 7 d of treatment with MP suspensions, the root elongation (Table 1) and the number of lateral roots (Figure 3b) of seedlings increased compared to the control, probably due to the adsorption of particles on the root surface causing the blockage of root hair cells and inhibiting effective water uptake [27], which in turn allowed the plants to mitigate this effect through root elongation and the differentiation of more lateral roots. This change in plant morphology is part of the plant’s growth adaptation strategy. Notably, the promotion effect of root elongation under the four types of particle exposure diminished with the photoaging of MPs (Table 1). The decrease in root elongation induced by photoaging is a result of plant defense against this external stress. It has also been reported [28,29] that the inhibition of cell elongation and local cell division is a common manifestation of stress-induced morphological changes in plants to mitigate toxicity effects. In contrast, pristine MP exposure showed no significant effect on the growth of seedling stems, and only the particles photoaged for 14 d caused the stems to exhibit an increase in elongation. This may be due to the fact that root tissues directly exposed to the MP suspension may present a more pronounced toxicity response, while the plant defense mechanism was able to prevent the transfer of this hazard to the above-ground parts to some extent. However, once the photoaging of MPs reached high levels, the self-protection mechanisms of the plant were not sufficient to defend against this hazard and resulted in the compromised growth of the above-ground parts. Several studies have also demonstrated that photoaging is able to enhance the phytotoxicity of MPs and thus increase the environmental risk of particles [16,30].

### 3.4. Nutrient Transport of Soluble Sugars in Roots and Stems

Sugar is one of the main components of plant carbon metabolism and nutrient delivery and usually serves as an intermediate product in the transport of carbon from source to sink [31]. Thus, it is crucial to understand the soluble sugar content in seedling tissues in terms of their growth and metabolic condition. Figure 3c,d shows the soluble sugar content of the roots and stems after 7 d of the four types of MP exposure for different photoaging times. It can be seen that the soluble sugar content of the four types of MPs on the stems decreased gradually with photoaging time, and the inhibition was most severe for PE MPs at the photoaging time of 14 d. In addition, PS and PET MPs had no significant effect on the soluble sugar content of the roots, while PA and PE MPs caused a pronounced decrease in their percentage content. The accumulation of soluble sugar content in the roots was as low as 0.26% and 0.28% under pristine PA and PE MP exposure, respectively. With the photoaging of MPs, the soluble sugar content in the roots of PA and PE MP treatment groups rebounded. Considering that sugar is a substrate for metabolism and a signaling molecule for various pathways [32], the inhibition of soluble gluconeogenesis indicates a possible impairment of carbon metabolism. Different types of MPs may have an effect on nutrients such as nitrogen and phosphorus [33,34], which may ultimately alter the accumulation of nutrients in plants. These results suggest that the accumulation of soluble sugars in sprouts is mainly influenced by the type of MP and that photoaging also interferes with the nutrient transport of sugar fractions from roots to stems.

### 3.5. Oxidative Stress Induced by Photoaging MPs

**Figure 4:**
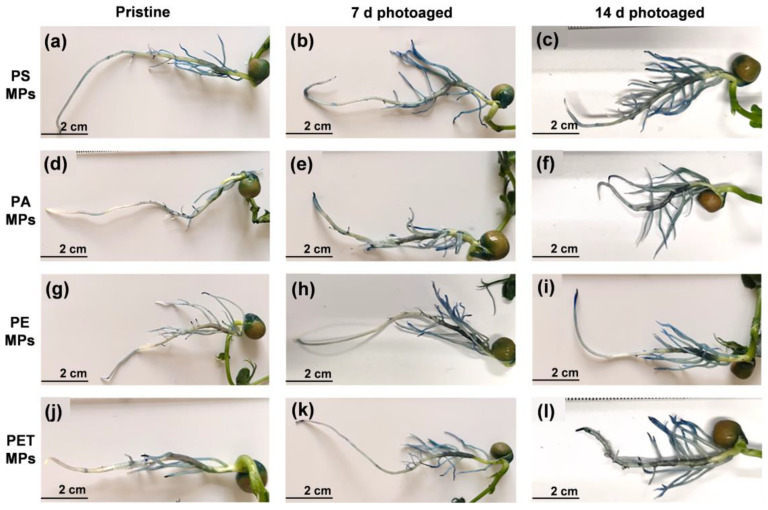
The growth status of seedling roots and Evans blue staining after 7 d of exposure to 100 mg L^−1^ particle suspensions of (**a**–**c**) PS, (**d**–**f**) PA, (**g**–**i**) PE, and (**j**–**l**) PET MPs photoaged for 0, 7, and 14 d.

**Figure 5:**
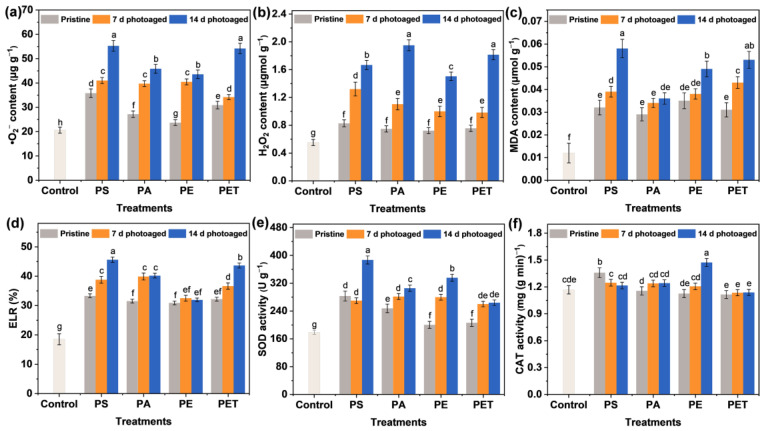
The concentration of (**a**) •O_2_^−^ and (**b**) hydrogen peroxide (H_2_O_2_), (**c**) malondialdehyde (MDA) content, (**d**) electrolyte leakage rate (ELR), and the activities of (**e**) superoxide dismutase (SOD) and (**f**) catalase (CAT) in roots after 7 d of exposure to 100 mg L^−1^ different MP suspensions. The number of samples for the repeated experiments is *n* = 3. Data are expressed as the mean ± SD. Different lowercase letters indicate significant differences between experimental groups (*p* < 0.05).

The plants are capable of generating massive amounts of ROS with a strong oxidative ability in the cells during environmental stress. These ROS can be involved in the substance metabolism of plants, thus inducing structural and functional damage to biofilms and even leading to protein and nucleic acid denaturation [35,36]. Therefore, it is essential to understand the oxidative damage (Figure 4 and Figure S1) and ROS accumulation (Figure 5) in plants under MP exposure in order to recognize MPs toxicity. The ROS contain singlet oxygen (^1^O_2_), •O_2_^−^, and H_2_O_2_ [37,38], where •O_2_^−^ is an anion radical generated during biological oxidation and electron transfer and plant cells generate H_2_O_2_ through processes such as disproportionation reactions of •O_2_^−^ catalyzed by SOD enzymes. More specifically, all pristine MPs induced an increase in •O_2_^−^ and H_2_O_2_ content in the roots compared to the control group (Figure 5a,b). Of these, the influence of PS and PET MPs on the induction of •O_2_^−^ production was more significant, resulting in the accumulation of •O_2_^−^ in the roots of up to 35.82 and 30.99 μg g^−1^, respectively. In contrast, there was little difference in the effect of the four types of pristine MPs on H_2_O_2_ production in the roots of seedlings. Furthermore, with the photoaging treatment of particles, exposure to four types of MP at 100 mg L^−1^ significantly raised the ROS production in the roots. The PS and PET MPs photoaged for 14 d were able to induce the generation of higher levels of •O_2_^−^, reaching 55.21 and 54.19 μg g^−1^, respectively, while PA and PET MPs induced a relatively high accumulation of H_2_O_2_, up to 1.67 and 1.82 μmol g^−1^, respectively. More seriously, both the •O_2_^−^ and H_2_O_2_ contents of seedlings roots were enhanced with the increasing photoaging time of MPs. These results suggest that MPs have the potential to induce ROS, as confirmed by the previous study [20], and this ability is affected by the MP type and photoaging time. This is consistent with the trend of ROS generation in the MP suspensions of each group, indicating that the accumulation of ROS in plant cells may be associated with the level of free radicals induced by MPs.

The massive accumulation of ROS in plant tissues may exacerbate lipid peroxidation and lead to irreversible oxidative damage. To observe MP-induced plant cell damage, Evans blue staining (Figure 4 and Figure S1) and MDA and ELR analysis (Figure 5c,d) were performed on seedlings from each group after 7 d of exposure. Evans blue fails to pass through normal cell membranes (Figure S1) and can enter the cells through the cell membrane and stain them blue only when cells are damaged [39]. The observation of the blue staining of roots (Figure 4) indicates the oxidative damage of the plants. It can be seen that the blue staining of the roots exposed to the pristine MPs was relatively weak and mainly distributed near the lateral roots, indicating that the damage was not severe. However, with increasing photoaging time, the roots of seedlings showed more and more significant damage. The blue staining appeared at the root tip and root elongation zone and gradually extended to all parts of the root tissue. Under the treatment of photoaged MPs, except for PE MPs, the other three types of MPs caused almost the whole root system to be stained blue. Notably, MDA content showed that photoaging of MPs enhanced the degree of lipid peroxidation in root cells, consistent with the trend of ELR changes, which also reflected the disruption of cell membrane integrity. Therefore, membrane damage and lipid peroxidation probably caused ROS generation, which further exacerbated the oxidative stress response in roots with the accumulation of ROS. It has also been reported [40] that the increase in •O_2_^−^ and •OH generated through the photoaging of MPs leads to an increase in the level of ROS in roots, ultimately inducing oxidative stress. Overall, photoaging is highly likely to enhance the environmental exposure and phytotoxicity of MPs.

### 3.6. Activation and Regulation of the Antioxidant System

**Figure 6:**
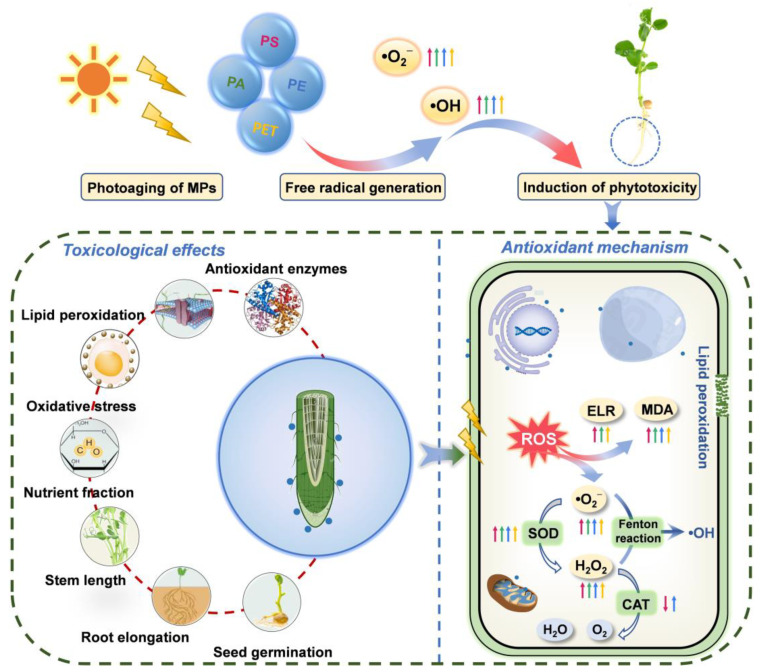
Schematic diagram of the mechanisms of physiological toxicity and antioxidant regulation induced by photoaging of MPs, including seed germination, root elongation, stem length, nutrient fraction, oxidative stress, lipid peroxidation, and antioxidant enzymes, as well as the scavenging mechanisms of the reactive oxygen species (ROS) via the antioxidant system. Straight arrows represent photoaged MPs; red arrows represent PS, green arrows represent PA, blue arrows represent PE, and orange arrows represent PET; arrows pointing up represent an increase, and arrows pointing down represent a decrease.

One of the regulatory systems for the scavenging of ROS in plants is the enzymatic antioxidant system, including SOD, CAT, peroxidase (POD), and glutathione reductase (GR). SOD is the first line of defense against ROS accumulation and membrane lipid peroxidation, mainly converting the highly toxic •O_2_^−^ into the less toxic H_2_O_2_. The immediate biological function of CAT is to promote the dismutation of H_2_O_2_ in cells to prevent the further production of the highly poisonous •OH. The modulation of the antioxidant system is able to reduce the phytotoxicity of MPs exposure. Specifically, PS photoaged for 14 d stimulated SOD activity (Figure 5e) to scavenge excess accumulated •O_2_^−^, thereby alleviating oxidative stress. Moreover, the roots in the PA treatment had the highest H_2_O_2_ content and CAT activity (Figure 5f), which was not significantly enhanced, presumably due to the rapid scavenging of •O_2_^−^ by SOD activity and the conversion to large amounts of less toxic H_2_O_2_. Similarly, the photoaged PE mainly activated CAT to scavenge excessive H_2_O_2_, whereas the increase in enzyme activities in the photoaged PET treatment was not apparent, indicating that PET induced stronger ROS accumulation in plants but was still within the plant tolerance range. Different types of MPs indeed showed differences in toxicity to the same plant, which may be related to the sensitivity and tolerance of plants and MPs types. In summary, the results emphasized that photoaging of MPs induced more severe phytotoxicity compared to pristine MPs. For 14 d photoaged MPs, PE exhibited the strongest inhibition of root elongation, PS caused the highest level of lipid peroxidation, while PET and PA presented a stronger tolerance. Colzi et al. [41] also investigated the toxicity mechanisms of PP, PE, PVC, and PET to *Cucurbita pepo* L. (pumpkin) and found differences in the antioxidant system and biochemical indicators, with the overall conclusion indicating that PVC was the most toxic and PE the least toxic. It has also been reported [30] that photoaging of MPs enhanced the adsorption capacity of the root epidermis through changes in physicochemical properties and induced potential biotoxicity on *Lepidium sativum* (garden cress) and *Sinapis alba* (white mustard) seedlings. Overall, the photoaging of MPs exacerbated the release of ROS from •O_2_^−^ and H_2_O_2_ and thus induced physiological toxicity and oxidative stress in plant cells. The activation of antioxidant systems played a critical role in resisting particle stress, improving the ROS scavenging ability and tolerance mechanisms in plants. The above schematic diagram on physiological toxicity and antioxidant modulation is presented in Figure 6.

## 4. Conclusions

This study explored the physiological toxicity, oxidative stress, and antioxidant regulation mechanisms of PS, PA, PE, PET, and their corresponding photoaged MPs on *Pisum sativum* L. seedlings. The results indicated that photoaging was able to promote the generation of •O_2_^−^ and •OH in MPs, and the ability to generate ROS was correlated with the type of particle. Moreover, different types of MPs were able to produce resistance and tolerance in seedlings and induce an increase in root and stem extension along with the number of lateral roots, but the promotion effect on root elongation was significantly inhibited through the photoaging of particles. The PA and PE MPs exhibited a remarkable inhibition of nutrient transport of soluble sugars in the root–stem system, thus possibly affecting the carbon metabolism of plants. In particular, the photoaging of PS and PE MPs induced excessive accumulations of •O_2_^−^ and H_2_O_2_ in roots, leading to pronounced lipid peroxidation and membrane damage in plant cells. The activation of the antioxidant system revealed that photoaged MPs regulated ROS scavenging capacity and stress tolerance mechanisms, emphasizing the toxicity modulation of different properties of particles on plant physiology. Overall, different types of MPs are able to induce corresponding toxicity effects, while photoaging promotes the generation of ROS in particles and further leads to enhanced phytotoxicity. Consequently, the photoaging behavior of MPs is an essential factor in understanding the environmental risk of plastics, and an in-depth knowledge of the environmental effects of MPs is critical for the toxicological study of plants.

## Figures and Tables

**Table 1:** The root elongation and stem length of seedlings after 7 d of exposure to 100 mg L^−1^ different MP suspensions. The number of samples for the repeated experiments is *n* = 3. Data are expressed as the mean ± SD. Different lowercase letters indicate significant differences between experimental groups (*p* < 0.05).

Plant Elongation	Photoaging Time	Control	PS MPs	PA MPs	PE MPs	PET MPs
Roots (cm)	Pristine	7.16 ± 0.36 ^def^	8.21 ± 0.33 ^ab^	7.96 ± 0.26 ^abcd^	8.57 ± 0.24 ^a^	8.15 ± 0.43 ^abc^
	7 d Photoaged		7.93 ± 0.40 ^abcd^	7.80 ± 0.32 ^abcd^	7.78 ± 0.39 ^abcde^	7.61 ± 0.35 ^bcdef^
	14 d Photoaged		7.38 ± 0.37 ^cdef^	6.95 ± 0.31 ^f^	7.35 ± 0.32 ^cdef^	7.08 ± 0.33 ^ef^
Stems (cm)	Pristine	5.42 ± 0.42 ^bc^	5.34 ± 0.36 ^c^	5.40 ± 0.38 ^c^	5.42 ± 0.31 ^bc^	5.63 ± 0.38 ^abc^
	7 d Photoaged		5.37 ± 0.40 ^c^	5.29 ± 0.41 ^c^	5.58 ± 0.42 ^abc^	6.15 ± 0.41 ^ab^
	14 d Photoaged		5.76 ± 0.49 ^c^	6.17 ± 0.45 ^a^	5.79 ± 0.52 ^abc^	6.23 ± 0.48 ^a^

## Data Availability

Information is available in the manuscript.

## Supplementary Materials

The following supporting information can be downloaded at: https://www.mdpi.com/article/10.3390/toxics11030242/s1, Detection of antioxidant enzyme activities in roots; Figure S1: Evans blue staining of seedling roots in the control group after 7 d of exposure. No significant blue staining occurred.
