# Supplementary material for: Physiological Toxicity and Antioxidant Mechanism of Photoaging Microplastics on Pisum sativum L. Seedlings
Source: Toxics. 2023 Mar 4;11(3):242. doi: 10.3390/toxics11030242 (PMC10056763; doi:10.3390/toxics11030242)
Supplement: Supplementary file 1 [file toxics-11-00242-s001.zip › toxics-2157861-supplementary.pdf]

Supplementary Materials

Physiological Toxicity and Antioxidant Mechanism of Photoaging Microplastics on *Pisum sativum* L. Seedlings

Meng'en Kang[1], *Yi Liu*[1], *Haoke Wang*[1], *Yuzhu Weng*[1], *Dongqing Gong*[1], *Xue Bai*[1,2,*]

[1]Key Laboratory of Integrated Regulation and Resource Development on Shallow Lake of Ministry of Education, College of Environment, Hohai University, Nanjing 210098, PR China

[2]Yangtze Institute for Conservation and Development, Hohai University, Nanjing 210098, PR China

*Corresponding author: Xue Bai;

Address: Xikang Road No.1, Gulou District, Nanjing, China, 210098

(College of Environment, Hohai University, Nanjing);

Email：baixue@hhu.edu.cn

Detection of antioxidant enzyme activities in roots

The activity of superoxide dismutase (SOD) was assayed by measuring its ability to inhibit the photochemical reduction of nitro blue tetrazolium (NBT). The reaction mixture was consisting of 50 mM phosphate buffer solution (PBS, pH 7.8), 20 μM riboflavin, 75 mM NBT, 13 mM methionine, and 0.1 mM ethylene diamine tetra acetic acid (EDTA). The mixture was irradiated under light tubes for 10 min. Blank and controls were also run in the same manner but without illumination and enzyme, respectively. The activity was assayed via spectrophotometer at 560 nm wavelength. The catalase (CAT) activity was assayed by taking a reaction solution inclosing PBS (50 mM, pH 7.8), H_2O_2 (300 mM), and enzyme extract. The absorbance was recorded at 240 nm for 30 s.

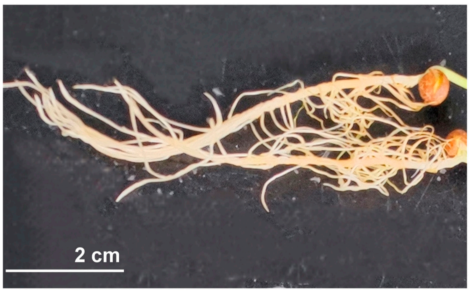

Figure S1. Evans blue staining of seedling roots in the control group after 7 d of exposure. No significant blue staining occurred.
